# Role of the Gastric Microbiome in Gastric Cancer: From Carcinogenesis to Treatment

**DOI:** 10.3389/fmicb.2021.641322

**Published:** 2021-03-15

**Authors:** Jinpu Yang, Xinxin Zhou, Xiaosun Liu, Zongxin Ling, Feng Ji

**Affiliations:** ^1^Department of Gastroenterology, The First Affiliated Hospital, Zhejiang University School of Medicine, Hangzhou, China; ^2^Department of Gastrointestinal Surgery, The First Affiliated Hospital, Zhejiang University School of Medicine, Hangzhou, China; ^3^Collaborative Innovation Center for Diagnosis and Treatment of Infectious Diseases, State Key Laboratory for Diagnosis and Treatment of Infectious Diseases, National Clinical Research Center for Infectious Diseases, The First Affiliated Hospital, School of Medicine, Zhejiang University, Hangzhou, China

**Keywords:** dysbiosis, gastric microbiome, gastric cancer, gastritis, *Helicobacter pylori*, peptic ulcers

## Abstract

The development of sequencing technology has expanded our knowledge of the human gastric microbiome, which is now known to play a critical role in the maintenance of homeostasis, while alterations in microbial community composition can promote the development of gastric diseases. Recently, carcinogenic effects of gastric microbiome have received increased attention. Gastric cancer (GC) is one of the most common malignancies worldwide with a high mortality rate. *Helicobacter pylori* is a well-recognized risk factor for GC. More than half of the global population is infected with *H. pylori*, which can modulate the acidity of the stomach to alter the gastric microbiome profile, leading to *H. pylori*-associated diseases. Moreover, there is increasing evidence that bacteria other than *H. pylori* and their metabolites also contribute to gastric carcinogenesis. Therefore, clarifying the contribution of the gastric microbiome to the development and progression of GC can lead to improvements in prevention, diagnosis, and treatment. In this review, we discuss the current state of knowledge regarding changes in the microbial composition of the stomach caused by *H. pylori* infection, the carcinogenic effects of *H. pylori* and non-*H. pylori* bacteria in GC, as well as the potential therapeutic role of gastric microbiome in *H. pylori* infection and GC.

## Introduction

Gastric cancer (GC) is the third leading cause of cancer death after lung and colorectum cancers, and accounts for 782,685 deaths worldwide each year (Bray et al., 2018). Risk factors for GC include *Helicobacter pylori* infection, age, high salt intake, low fruit and vegetables intake, alcohol consumption, and smoking (Parsonnet et al., 1991; Jakszyn and Gonzalez, 2006; Anderson et al., 2010; Butt et al., 2019; Kumar et al., 2020; Li et al., 2021). The early stage of disease is asymptomatic or has non-specific symptoms (Luan et al., 2020); therefore, most patients are not diagnosed until an advanced stage.

*Helicobacter pylori* infection is common, affecting >50% of the global population with a higher incidence in developing countries (Hooi et al., 2017). The prevalence of *H. pylori* infection varies by age, ethnicity and living conditions (Seyda et al., 2007; Laszewicz et al., 2014; Alberts et al., 2020), and most cases occur in childhood (Banatvala et al., 1993). Only a small percentage of people develop pathological conditions related to *H. pylori* infection such as chronic gastritis, peptic ulcers, gastric adenocarcinoma, and gastric mucosa-associated lymphoid tissue (MALT) lymphoma (Wang et al., 2014). Chronic gastritis is the early presentation of persistent inflammation caused by *H. pylori* infection. As the condition progresses, injury to gastric epithelial cells can lead to the development of GC (Kidane, 2018). *H. pylori* has been listed as a Type I carcinogen by the World Health Organization (WHO) (International Agency for Research on Cancer, 1994). Therefore, detecting and eradicating *H. pylori* in the early phase of infection can prevent GC and other gastrointestinal diseases.

Microbiomes are complex microbial communities composed of bacteria, fungi, and viruses that reside in distinct habitats in the human body (Human Microbiome Project Consortium, 2012). The colon is among the most widely studied human microbial ecosystems as it contains the largest population of microorganisms (Villéger et al., 2019). The human microbiome and its metabolites have both physiologic and pathologic functions in homeostasis maintenance and disease development (Gilbert et al., 2018). In recent decades, there has been increasing interest in the relationship between the human microbiome and diseases. Despite the evidence that disruption the balance between microbiome and host in the stomach can promote the development of GC, the mechanism is not clearly understood.

To gain a better understanding of the relationship between the gastric microbiome and gastric carcinogenesis, here we provide an update on the gastric microbiome in healthy state and *H. pylori*-associated pathological conditions, including gastritis, peptic ulcer disease, and GC. Especially, we focus on the possible mechanisms of gastric microbiome in the development of GC. The potential therapeutic role of gastric microbiome in *H. pylori* infection and GC is also discussed.

## The Human Gastric Microbiome

### The Healthy Gastric Microbiome

The stomach was previously thought to be a sterile organ because of its strongly acidic environment. However, the discovery of *H. pylori* in the stomach of patients with gastritis and peptic ulcers by Marshall and Warren in 1982 refuted this notion (Marshall and Warren, 1984). Classical methods for studying the human gastric microbiome relied on microbiologic techniques including culture, isolation, and identification. However, as only a small number of gastric microorganisms can be grown under standard culture conditions, most microorganisms cannot be identified by this approach. The microorganisms most frequently isolated from the human stomach by culture-dependent methods were *Veillonella*, *Lactobacillus*, and *Clostridium* spp. (Zilberstein et al., 2007). Additionally, a large number of taxa have since been detected with newer technologies such as random shotgun sequencing, microarrays, and next-generation sequencing. The microbial load of the stomach is approximately 10^2^–10^4^ colony−forming units (CFU)/ml, which is much lower than that of the intestine (10^10^–10^12^ CFU/ml) (Delgado et al., 2013). *Proteobacteria*, *Firmicutes*, *Bacteroidetes*, *Actinobacteria*, and *Fusobacteria* are the most highly represented phyla in gastric mucosa under normal conditions (Bik et al., 2006; Delgado et al., 2013; Liu et al., 2019a). Human gastric juice also has a diverse microbial community that is distinct from that of the gastric mucosa (Sung et al., 2016): the former is dominated by *Firmicutes*, *Actinobacteria* and *Bacteroidetes*, while the latter mainly includes *Proteobacteria* and *Firmicutes* (Bik et al., 2006; Nardone and Compare, 2015; Sung et al., 2016). Additionally, bacteria present in the oral cavity and duodenum such as *Veillonella, Lactobacillus*, and *Clostridium* can transiently colonize the stomach (Zilberstein et al., 2007; Nardone and Compare, 2015). Thus, the microbial community in gastric juice may not be representative of the gastric microbiome as a whole.

The specific mechanisms contributing to inter-individual variations in gastric microbiome composition are not well understood. Microbiome composition is affected by childbirth delivery mode (in infants), age, sex, ethnicity, diet, lifestyle, geography, use of antibiotics, use of proton pump inhibitors (PPI) or histamine H2 receptor antagonists, and the presence of *H. pylori* (Tsuda et al., 2015; Bokulich et al., 2016; Haro et al., 2016; Lloyd-Price et al., 2016; Yang et al., 2016; Nardone et al., 2017). The acidic environment of the healthy stomach prevents the overproliferation of bacteria and reduces the risk of infection (Howden and Hunt, 1987). Long-term treatment with PPI or H2 antagonists reduces gastric acid secretion, leading to bacterial overgrowth (Alarcón et al., 2017). Antibiotic usage, immunosuppression, and gastric fluid pH > 4 were found to be associated with reduced bacterial diversity in the stomach (Von Rosenvinge et al., 2013). Interestingly, a study of the gastric microbiome in twins showed that genetic background had no influence on gastric microbial community structure (Dong et al., 2017); similar findings were also reported for different niches of the human body (Lee et al., 2011; Lee J.E. et al., 2013).

### Effects of *H. pylori* Infection on the Gastric Microbiome

The interactions between *H. pylori* and stomach-resident bacteria are not fully known. *H. pylori* is the predominant bacterium in the stomach of *H. pylori*-infected patients (Yu et al., 2017). However, low numbers of *H. pylori* have been detected by broad-range polymerase chain reaction and 16S rDNA sequence analysis in patients who were found to be negative for *H. pylori* infection by traditional methods such as histopathology, rapid urease test, serologic analysis, and culture (Bik et al., 2006). Thus, pyrosequencing has been used to define a cutoff value for *H. pylori* infection in human gastric samples (Kim et al., 2015).

Most *H. pylori* strains can modulate the gastric environment, thus altering the habitat of resident microorganisms (Mitchell et al., 2017). Alterations in gastric microbiome profile can increase the risk for developing GC (Liu et al., 2019a). A barcoded pyrosequencing analysis of 6 Swedish patients without or with *H. pylori* infection found that those who were *H. pylori*-negative had a more diverse gastric microbiome than patients testing positive (Andersson et al., 2008). In another study, children infected with *H. pylori* had a lower alpha diversity than *H. pylori*-negative children (Llorca et al., 2017). It was also reported that eradication of *H. pylori* increased microbial diversity in the stomach (Li et al., 2017). An examination of patients at different histologic stages of gastric carcinogenesis (gastritis, gastric intestinal metaplasia, and GC) revealed an inverse relationship between *H. pylori* abundance and microbial diversity in non-cancer gastric biopsies, but GC was associated with a lower diversity compared to other samples with similar *H. pylori* abundance; the difference was abrogated by antibiotic treatment (Li et al., 2017). A pyrosequencing analysis of the gastric microbiome demonstrated that *Proteobacteria*, *Firmicutes*, *Actinobacteria*, *Bacteroidetes*, and *Fusobacteria* were the major phyla in both *H. pylori*-negative and -positive patients (Jo et al., 2016). Similarly, *Proteobacteria*, *Firmicutes*, *Bacteroidetes*, and *Actinobacteria* were the most abundant phyla in a pediatric population (51 pediatric patients; 18 positive and 33 negative for *H. pylori*), but their relative proportions differed between *H. pylori*-positive vs. -negative patients (Llorca et al., 2017). Another study found that *Proteobacteria*, *Spirochetes*, and *Acidobacteria* were highly represented in *H. pylori*-positive patients whereas *Actinobacteria*, *Bacteroidetes*, and *Firmicutes* were detected at low levels (Maldonado-Contreras et al., 2011).

## *Helicobacter pylori* Infection and Gastric Microbiome in Gastric Diseases

### Gastritis

Chronic atrophic gastritis is a gastric premalignant condition (Sugano et al., 2015). Most cases of chronic atrophic gastritis are caused by persistent *H. pylori* infection (Sipponen and Maaroos, 2015), which triggers an inflammatory response that has various effects on gastric epithelial cells such as disruption of the gastric barrier (Gajewski et al., 2016), induction of apoptosis (Wan et al., 2016), and stimulation of proinflammatory cytokine secretion (Peek et al., 1995). Microbial composition in the stomach was shown to be altered in patients with chronic atrophic gastritis, with *Helicobacteraceae*, *Streptococcaceae*, *Fusobacteriaceae*, and *Prevotellaceae* as the major taxa (Parsons et al., 2017). Moreover, the abundance of *Tannerella*, *Treponema*, and *Prevotella* spp. was shown to be reduced in atrophic gastritis patients compared to healthy controls (Zhang et al., 2019), and a recent study showed that several pathways were significantly altered in patients with *H. pylori*-induced atrophic gastritis, including underrepresented (e.g., succinate dehydrogenase and tagaturonate reductase) and overrepresented (e.g., fumarate reductase, ketol-acid reductor isomerase, glycolate oxidase, and alanine dehydrogenase) pathways (Parsons et al., 2017). *H. pylori* infection stimulates the production and release of proinflammatory factors (Outlioua et al., 2020): gastric mucosal interleukin (IL)-8 level was correlated with the severity of the *H. pylori*-induced atrophic gastritis (Xuan et al., 2005; Lee K.E. et al., 2013); serum tumor necrosis factor (TNF)-α level was found to be related to the degree of chronic inflammation and neutrophil infiltration (Siregar et al., 2015); and serum vascular endothelial growth factor (VEGF) level was linked to the severity of gastric lesions in patients with gastritis (Siregar et al., 2015).

### Peptic Ulcer Disease

Peptic ulcer disease is defined as acid-induced peptic injury of the mucosa reaching the submucosa (Lanas and Chan, 2017). Most cases of peptic ulcer disease are caused by *H. pylori* infection and use of non-steroidal anti-inflammatory drugs (NSAIDs), and other risk factors include gastrinoma, smoking, and use of other medications (Bernersen et al., 1996; Kavitt et al., 2019). *H. pylori* infection and the use of NSAIDs were shown to synergistically promote the development of peptic ulcer disease (Huang et al., 2002). However, only a small proportion of patients with *H. pylori* infection or who use NSAIDs progress to peptic ulcer disease, suggesting that decreased mucosal resistance to bacterial virulence factors and drug toxicity contribute to its pathogenesis (Lanas and Chan, 2017). *H. pylori* eradication therapy was shown to reduce the rate of recurrence (Tomita et al., 2002; Feder et al., 2018). A matrix-assisted laser desorption ionization time-of-flight mass spectrometry analysis showed that non-*H. pylori* bacteria were more abundant in patients with non-ulcer dyspepsia than in those with gastric ulcers, and the predominant non-*H. pylori* bacteria in *H. pylori*-positive patients were *Streptococcus*, *Neisseria*, *Rothia*, and *Staphylococcus* (Hu et al., 2012). Another study in Malaysian patients showed similar microbiome profiles in *H. pylori*-positive and -negative patients as well as an association between the isolation of *Streptococci* and peptic ulcer disease, suggesting that *Streptococci* colonize the stomach and that their interactions with *H. pylori* contribute to the development of peptic ulcer disease (Khosravi et al., 2014).

### Gastric Cancer

*Helicobacter pylori* infection leads to persistent inflammation of gastric mucosa that causes changes in the cell cycle of gastric epithelial cells, which eventually result in atrophy of the glands, intestinal metaplasia, and GC (Correa, 1992). In addition to *H. pylori*, other microorganisms in the stomach have been implicated in gastric carcinogenesis (Table 1). Therefore, clarifying their distribution and functions can serve as a basis for the development of new therapeutic strategies.

**TABLE 1:** Studies on dysbiosis of gastric microbiome in gastric cancer.

**Authors**	**Year**	**Sample size**	**Result**
Aviles-Jimenez et al.	2014	• 5 patients each with non-atrophic gastritis, intestinal metaplasia, and gastric cancer of the intestinal type	• Bacterial diversity steadily decreased from non-atrophic gastritis to intestinal metaplasia to gastric cancer • A significant microbiota difference was observed between non-atrophic gastritis and gastric cancer
Wang et al.	2016	• 212 patients with chronic gastritis and 103 patients with gastric cancer	• The amount of bacteria in gastric mucosa was higher in *Helicobacter pylori*-infected patients compared with those uninfected • An increased bacterial load was detected in gastric cancer compared with chronic gastritis • Five genera of bacteria were enriched in gastric cancer. Including *Lactobacillus*, *Escherichia–Shigella*, *Nitrospirae*, *Burkholderia fungorum*, and *Lachnospiraceae*
Jo et al.	2016	• 63 antral mucosal and 18 corpus mucosal samples	• The number of nitrosating or nitrate-reducing bacteria (NB) other than *H. pylori* (non-HP-NB) was two times higher in the cancer groups than in the control groups, but it did not reach statistical significance
Yu et al.	2017	• 160 gastric cancer patients with 80 from China and 80 from Mexico	• *Helicobacter pylori* is the most abundant member of gastric microbiota in both Chinese and Mexican samples, followed by oral-associated bacteria
Castaño-Rodríguez et al.	2017	• 12 patients with gastric cancer, 20 patients with functional dyspepsia	• Increased richness and phylogenetic diversity but not Shannon’s diversity was found in gastric cancer as compared to controls • Several bacterial taxa were enriched in gastric cancer, including *Lactococcus*, *Veilonella*, and *Fusobacteriaceae*
Hsieh et al.	2018	• 9 patients with gastritis, 7 patients with intestinal metaplasia, 11 patients with gastric cancer	• The frequency and abundance of *H. pylori* were significantly lower in the cancer group • *Clostridium*, *Fusobacterium*, and *Lactobacillus* species were frequently abundant in patients with gastric cancer
Ferreira et al.	2018	• 54 patients with gastric carcinoma and 81 patients with chronic gastritis	• The gastric carcinoma microbiota was characterized by reduced microbial diversity, by decreased abundance of *Helicobacter* and by the enrichment of other bacterial genera, mostly represented by intestinal commensals
Coker et al.	2018	• 21 superficial gastritis, 23 atrophic gastritis, 17 intestinal metaplasia and 20 gastric cancer subjects	• Significant mucosa microbial dysbiosis was observed in intestinal metaplasia and gastric cancer subjects, with significant enrichment of 21 and depletion of 10 bacterial taxa in gastric cancer compared with superficial gastritis • *Peptostreptococcus stomatis*, *Streptococcus anginosus*, *Parvimonas micra*, *Slackia exigua*, and *Dialister pneumosintes* had significant centralities in the gastric cancer ecological network
Liu et al.	2019a	• 276 gastric cancer patients without preoperative chemotherapy • 230 normal, 247 peritumoral and 229 tumoral tissues	• *H. pylori*, *Prevotella copri* and *Bacteroides uniformis* were significantly decreased in tumoral microhabitat • *Prevotella melaninogenica*, *Streptococcus anginosus*, and *Propionibacterium acnes* were increased in tumoral microhabitat
Ling et al.	2019	• 64 gastric cancer patients without preoperative chemotherapy • 59 tumoral tissues, 61 peritumoral tissues, and 60 normal tissues	• The diversity, composition and function of gastric mucosal microbiota also changed more significantly in tumoral tissues than those in normal and peritumoral ones • *Stenotrophomonas* and *Selenomonas* were positively correlated with BDCA2 + pDCs and Foxp3 + Tregs, respectively • *Comamonas* and *Gaiella* were negatively correlated with BDCA2 + pDCs and Foxp3 + Tregs, respectively
Chen et al.	2019	• 62 pairs of matched gastric cancer tissues and adjacent non-cancerous tissues	• Microbial richness and diversity were increased in cancerous tissues • The bacterial taxa enriched in the cancer samples were predominantly represented by oral bacteria (such as *Peptostreptococcus*, *Streptococcus*, and *Fusobacterium*), while lactic acid-producing bacteria (such as *Lactococcus lactis* and *Lactobacillus brevis*) were more abundant in adjacent non-tumor tissues
Gunathilake et al.	2019	• 268 gastric cancer patients and 288 controls	• *Helicobacter pylori*, P*ropionibacterium acnes* and *Prevotella copri* are strong risk factors, whereas *Lactococcus lactis* is a protective factor, for gastric cancer development in Koreans
Gantuya et al.	2020	• 48 gastric cancer and 120 non-cancer patients (20 normal gastric mucosa, 20 gastritis, 40 with atrophy and 40 intestinal metaplasia)	• The highest overall bacterial alpha diversity metrics were observed in the control group, followed by the intestinal metaplasia and cancer groups. The gastritis and atrophy groups had the least diversity
Wang Z. et al.	2020	• 30 healthy controls, 21 non-atrophic chronic gastritis, 27 gastric intestinal metaplasia, 25 intraepithelial neoplasia, and 29 gastric cancer patients	• The bacterial diversity reduced progressively from non-atrophic chronic gastritis, through intestinal metaplasia, intraepithelial neoplasia to gastric cancer
Wang L. et al.	2020	• 60 patients with chronic gastritis, 30 with early gastric cancer, and 30 with advanced gastric cancer	• The results demonstrated significant differences in the microbial profile and composition between early gastric cancer and advanced gastric cancer
Ravegnini et al.	2020	• 10 adenocarcinoma and 10 signet-ring cell carcinoma and their paired non-tumor counterparts	• Signet-ring cell carcinomas were significantly enriched in the phyla *Fusobacteria*, *Bacteroidetes*, *Patescibacteria*, whereas in the adenocarcinoma type, *Proteobacteria* and *Acidobacteria* phyla were found

Dysbiosis is defined as the compositional and functional alterations of the microbiome (Levy et al., 2017). In spite of the growing number of studies exploring the microbial dysbiosis during gastric carcinogenesis, there is still no consensus in terms of the alteration pattern of the gastric microbiome. There are some studies suggesting that the microbial diversity is markedly reduced in inflammatory diseases and cancer (Ahn et al., 2013; Aviles-Jimenez et al., 2014; Gong et al., 2016). Certainly, GC is no exception (Ferreira et al., 2018). However, some studies indicated that the richness and diversity of gastric microbiome in GC tissues were increased compared to control tissues (Castaño-Rodríguez et al., 2017; Chen et al., 2019). It has been reported that the bacterial richness and diversity decreased gradually from healthy control, through non-atrophic chronic gastritis, intestinal metaplasia, IN to GC (Wang Z. et al., 2020). However, another study showed that the bacterial diversity reduced gradually from normal, intestinal metaplasia, GC, gastritis to atrophy (Gantuya et al., 2020). Moreover, microbial diversity was found to be increased in advanced-stage GC compared to the early stage, which did not differ significantly from that in chronic gastritis (Wang L. et al., 2020). *Novosphingobium*, *Ralstonia*, *Ochrobactrum*, *Anoxybacillus*, and *Pseudoxanthomonas* were enriched in early GC whereas *Burkholderia*, *Tsukamurella*, *Uruburuella*, and *Salinivibrio* were more abundant in advanced as compared to early GC (Wang L. et al., 2020). However, another study reported that there was no significant difference in microbial community composition between early- and late-stage GC, while microbial richness decreased from normal to peritumoral to tumoral tissues (Liu et al., 2019a). Additionally, *Prevotella copri* and *Bacteroides uniformis* were reduced whereas *Prevotella melaninogenica*, *Streptococcus anginosus*, and *Propionibacterium acnes* were enriched in tumor tissue compared to normal and peritumoral tissues (Liu et al., 2019a). A recent study gave insight into the microbial composition in different subtypes of GC. *Fusobacteria*, *Bacteroidetes*, *Patescibacteria* were enriched in signet-ring cell carcinoma, whereas *Proteobacteria* and *Acidobacteria* were enriched in adenocarcinoma (Ravegnini et al., 2020).

Dysbiosis of the oral microbiome has been linked to inflammatory bowel disease, colorectal cancer, and pancreatic cancer (Nakatsu et al., 2015; Atarashi et al., 2017; Gaiser et al., 2019). The abundance of oral microbiota including *Peptostreptococcus*, *Streptococcus*, and *Fusobacterium* was shown to be higher in GC samples than in adjacent non-tumor samples (Chen et al., 2019). Similarly, an investigation of the gastric microbiome in superficial gastritis, atrophic gastritis, intestinal metaplasia, and GC by 16S rRNA gene sequencing revealed that oral bacteria such as *Peptostreptococcus stomatis*, *S. anginosus*, *Parvimonas micra*, *Slackia exigua*, and *Dialister pneumosintes* were enriched in GC compared to tissue samples from precancerous stages (Coker et al., 2018). It is possible that alterations in the acidic environment of the stomach in GC enable colonization by oral bacteria (Chen et al., 2019). However, further studies are needed to clarify the role of the oral microbiome in gastric carcinogenesis.

*Lactobacillus* is a major genus in the gut microbiome and is used to relieve various gastrointestinal conditions (Salazar-Lindo et al., 2007). Lactic acid production has several biologically important functions including immunomodulation and anti-inflammatory and anti-cancer effects (Han et al., 2015; Ghosh et al., 2019). However, lactic acid-producing bacteria also play a role in gastric carcinogenesis (Vinasco et al., 2019). A study conducted in Taiwan found that the abundance of *Lactobacillus* was increased in patients with GC compared to those with gastritis or intestinal metaplasia (Hsieh et al., 2018), which is partly consistent with other reports (Castaño-Rodríguez et al., 2017; Ferreira et al., 2018). Another study examining the abundance of lactic acid-producing bacteria in the tumor microenvironment reported that lactic acid-producing bacteria such as *Lactococcus lactis* and *Lactobacillus brevis* were enriched in adjacent non-tumor tissue (Chen et al., 2019). Animal experiment also revealed the potential carcinogenic role of *Lactobacillales* in GC (Bali et al., 2021).

Microbial interactions determine the microbiome homeostasis and influence the disease-associated microenvironment. The reduced complexity of the microbial interaction network in GC was attributed to the lower abundance of *H. pylori* (Liu et al., 2019a; Wang L. et al., 2020). Other studies have suggested that the interaction network is more complex in GC, which may be the result of reduced *H. pylori* abundance accompanied by an increased abundance of other microorganisms (Ferreira et al., 2018; Chen et al., 2019).

## Mechanisms of Carcinogenesis

Mechanisms employed by gastric microbiota to promote gastric carcinogenesis include activation of inflammation, modulation of the host immune response, regulation of tumor growth and angiogenesis, production of microbial metabolites, and induction of DNA damage (Ou et al., 2019; Lee et al., 2020; Leite et al., 2020; Sierra et al., 2020).

### *Helicobacter pylori* and Gastric Carcinogenesis

*Helicobacter pylori* is a Gram-negative spiral-shaped bacterium with urease, catalase, and oxidase activities (Kusters et al., 2006). It is thought that *H. pylori* colonized modern humans over 50,000 years ago and has evolved to resist the harsh acidic environment of the human stomach (Linz et al., 2007). The flagellum and spiral shape of *H. pylori* allow it to achieve primary infection and colonize the gastric mucosa (Sycuro et al., 2012; Gu, 2017). *H. pylori* urease transforms urea into ammonia to neutralize stomach acid (Scott et al., 2010). The functional and structural features of *H. pylori* allow it to traverse the gastric mucus, form a protected niche adjacent to the surface of the gastric epithelium, and deliver its products to host cells, which is critical for its survival in the human stomach and evasion of the host immune response, and for promoting disease development (Olofsson et al., 2014; Zhang et al., 2017; Pachathundikandi et al., 2019). Genetic diversity – a prominent feature of *H. pylori* strains that arises from point mutations and intra-/intergenomic recombination (Kraft and Suerbaum, 2005) – was shown to be correlated with the pathogenicity of *H. pylori* strains and influence the risk of malignant transformation (Yadegar et al., 2019).

#### *Helicobacter pylori* Virulence Factors

*Helicobacter pylori* strains have multiple virulence factors that directly or indirectly influence the risk of GC development (Ansari and Yamaoka, 2019). One of the most important of these virulence factors is VacA, which was initially identified by its ability to induce vacuolation in epithelial cells (Cover and Blaser, 1992). VacA is a multifunctional toxin that exhibits effects in different host cell types (e.g., gastric epithelial cells, antigen-presenting cells, phagocytic cells, mast cells, and T cells) (Supajatura et al., 2002; Torres et al., 2007; Manente et al., 2008; Altobelli et al., 2019). Aside from vacuolation, VacA impairs host gastric epithelial cells in a variety of ways – for example, by increasing mitochondrial membrane permeability, disrupting endocytic trafficking, and inducting apoptosis (Willhite and Blanke, 2004; Gauthier et al., 2005; Matsumoto et al., 2011). Additionally, VacA modulates the host immune response by inhibiting the activation and proliferation of immune cells and stimulating the production of proinflammatory cytokines (e.g., TNF-α and IL-6) by mast cells to promote the development of *H. pylori*-associated gastritis, peptic ulcer disease and GC (Supajatura et al., 2002; Torres et al., 2007).

Another virulence factor associated with the development of GC is CagA, which is encoded by the *cagA* gene located at one end of the cag pathogenicity island (*cag* PAI) (Hatakeyama, 2017). The *cag* PAI also encodes the type IV bacterial secretion system (T4SS) (Knorr et al., 2019), which forms a complex that delivers CagA from adherent *H. pylori* to host cells across the outer and inner bacterial membranes (Chung et al., 2019). After translocation, CagA can act on gastric epithelial cells to promote carcinogenesis by promoting inflammation, inducing proliferation, inhibiting apoptosis, disrupting cell–cell junctions, and causing the loss of cell polarity (Bagnoli et al., 2005; Buti et al., 2011, 2020; Yang et al., 2018).

#### *Helicobacter pylori* and Immune Response

*Helicobacter pylori* infection can stimulate both innate and adaptive immune responses (Bimczok et al., 2010; Freire de Melo et al., 2014). *H. pylori* violence factors activate the host immune response (Karkhah et al., 2019). The pattern recognition receptors (PRRs) of host cells recognize *H. pylori* pathogen-associated molecular patterns (PAMPs), triggering the initial stage of the innate immune response (Uno et al., 2007). Toll-like receptors (TLRs) are major components of PRRs that can bind to lipopolysaccharide, lipoproteins, lipoteichoic acid, double-stranded RNA, flagellin, unmethylated nucleic acids, and CpG repeats of *H. pylori* (Satoh and Akira, 2016). After recognizing PAMPs, TLRs activate NF-κB, interferon regulatory factor (IRF), and activator protein (AP)-1 to stimulate the expression of inflammatory mediators such as interferon (IFN)-γ, IL-1, IL-2, IL-6, IL-8, IL-12, and TNF-α (Kawasaki and Kawai, 2014; Nejati et al., 2018). Interestingly, *H. pylori* can evade the recognition by host PRRs in the innate immune response, thereby ensuring its long-term survival (Sun et al., 2013; Devi et al., 2015). In adaptive immunity, cluster of differentiation (CD)4 + T cells are the main mediators of the host immune response to *H. pylori* infection (Karkhah et al., 2019). CD4 + T cells were more abundant in GC samples compared to peritumoral and normal tissue samples, whereas CD8 + T cells showed the opposite distribution pattern (Huang et al., 2014). The release of inflammatory mediators induced by *H. pylori* virulence factors during the innate immune response activates T helper (Th)1/Th17 cell responses and stimulates the production of IFN-γ, IL-17, and TNF-α (Bimczok et al., 2010; Beigier-Bompadre et al., 2011). Thus, Th1/Th17 cells mediate the inflammatory response in *H. pylori*-infected patients (Bimczok et al., 2010; Beigier-Bompadre et al., 2011). Inflammation causes the loss of acid-secreting parietal cells, leading to an increase in pH in the stomach, which results in the decreased abundance of *H. pylori* and increased colonization by other bacteria (Pereira-Marques et al., 2019). *H. pylori* and the chronic inflammation that it induces enhance the production of reactive oxygen species (ROS) and reactive nitrogen species (RNS), which cause DNA damage such as point mutations and double-strand DNA breaks, dysregulate signal transduction pathways, and induce apoptosis or autophagy of gastric epithelial cells (Handa et al., 2010; Toller et al., 2011; Shimizu et al., 2017). The DNA repair system was found to be impaired in *H. pylori*-positive gastric epithelial cells (Han et al., 2020). Thus, *H. pylori* may promote gastric carcinogenesis by causing genetic instability in host cells. Additionally, ROS induce DNA mutations in *H. pylori* that allow it to adapt to the host environment (Gobert and Wilson, 2017).

In addition to its impact on effector T cells, *H. pylori* activates immunosuppressive responses in the host. Regulatory T cells (Tregs) inhibit aberrant or excessive immune responses that can be damaging to the host (Liu et al., 2015). Forkhead box protein (Foxp)3 + Tregs are the most important regulators of immune suppression (Deng et al., 2010). Mutation of Foxp3 is associated with severe autoimmune disease in both humans and animal models (Colobran et al., 2016; Ujiie and Shevach, 2016). An increased number of Foxp3 + Tregs was observed in tumor and peritumoral samples (Ling et al., 2019), and increased expression of Foxp3 in tumor-infiltrating Tregs suppressed T cell proliferation and was associated with tumor-node-metastasis stage in GC patients (Yuan et al., 2010). In *H. pylori*-positive individuals, the impaired immune response of CD4 + memory T cells to *H. pylori* antigens was rescued by depletion of Foxp3 + Tregs (Lundgren et al., 2003, 2005). Meanwhile, in mice infected with *H. pylori*, Treg deficiency resulted in severe gastric inflammation and reduced colonization by *H. pylori* (Rad et al., 2006). Foxp3 + Tregs can be subdivided into inducible costimulator (ICOS) + and ICOS-cells (Ito et al., 2008). ICOS + Foxp3 + Tregs secrete IL-10 and transforming growth factor (TGF)-β to suppress the function of dendritic cells (DCs) and T cells, respectively (Ito et al., 2008), and have been linked to poor clinical outcome in GC (Liu et al., 2019b). Thus, the current evidence suggests that Tregs enhance *H. pylori*-induced inflammation and promote the development of GC by suppressing host immune responses.

DCs function as a bridge between innate and adaptive immunity. The maturation status of DCs determines their immune function (enhancing immunity or promoting immunologic tolerance) and clinical outcomes in cancer (Karthaus et al., 2012). As one DCs subtype, plasmacytoid (p)DCs promote immunologic tolerance and tumor development (Hartmann et al., 2003; Perrot et al., 2007). The number of blood DC antigen (BDCA)2 + pDCs was found to be increased in tumor and peritumoral samples (Ling et al., 2019), which predicted a poor prognosis in patients with GC (Liu et al., 2019b). It has been suggested that tumor-infiltrating pDCs induce the activation and expansion of ICOS + Foxp3 + Treg cells to achieve immune suppression (Conrad et al., 2012).

### Other Gastric Bacteria and Gastric Carcinogenesis

**FIGURE 1:**
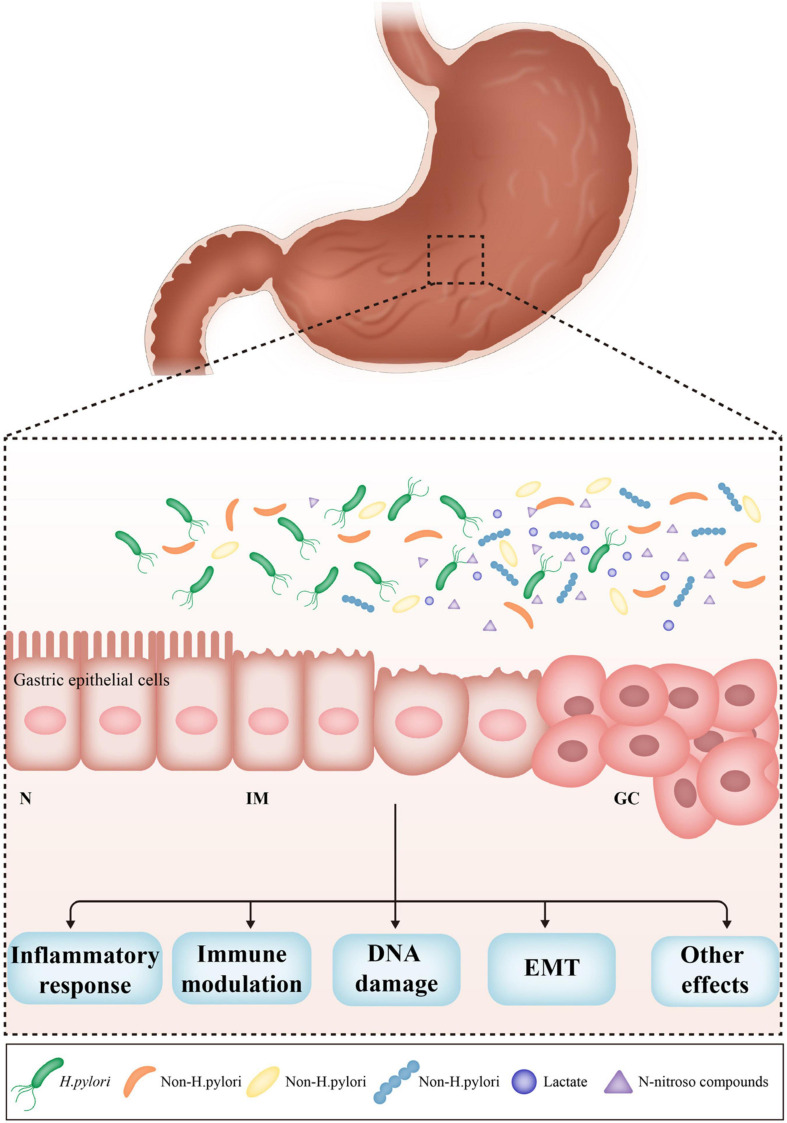
The effects of non-*Helicobacter pylori* bacteria on gastric carcinogenesis. *H. pylori* infection triggers the inflammatory response, which causes the loss of acid-secreting parietal cells, leading to the increased pH in the stomach. The alteration of acidic environment of the stomach allows colonization of other bacteria, subsequently results in dysbiosis of gastric microbiome. Non-*H. pylori* bacteria promote gastric carcinogenesis through their own characteristics and microbial metabolites, such as N-nitroso compounds and lactate. The main possible mechanisms include induction of inflammatory response, modulation of immune response, induction of DNA damage, and promotion of EMT. N, normal; IM, intestinal metaplasia; GC, gastric cancer; EMT, epithelial–mesenchymal transition.

Non-*H. pylori* bacteria also contribute to gastric carcinogenesis. A prospective randomized controlled trial demonstrated that the incidence of GC was similar in patients receiving *H. pylori* eradication therapy vs. a placebo over a period of 7.5 years in a high-risk region of China (Wong et al., 2004). *H. pylori* monoassociation accelerated the progression of atrophic gastritis and gastrointestinal intraepithelial neoplasia (GIN) in germ-free insulin-gastrin mice, but induced less severe gastric lesions and delayed the onset of GIN compared to mice harboring a complex microbiome. These results indicate that some microorganisms in the stomach play a critical role in the development of GC (Figure 1). However, it is unclear which non-*H. pylori* bacteria dominate this process, and the pathogenic mechanisms have yet to be established.

#### Non-*H. pylori* Bacteria That Promote Gastric Carcinogenesis

A recent case-control study conducted in Korean investigating gastric microbiome profiles found that patients with GC had a higher abundance of *P. acnes* and *P. copri* than control subjects, suggesting that the presence of these species increases the risk of GC (Gunathilake et al., 2019). *P. acnes* and its products may trigger corpus-dominant lymphocytic gastritis via activation of the natural killer group 2 member D (NKG2D) system and secretion of the proinflammatory cytokine IL−15 (Montalban-Arques et al., 2016). In fact, NKG2D ligand (NKG2DL) was shown to be upregulated in tumor cells (Guerra et al., 2008), and the NKG2D–NKG2DL system and IL−15 have been implicated in carcinogenesis (Oppenheim et al., 2005). However, *H. pylori* is unable to activate NKG2D–NKG2DL system and IL−15 expression (Montalban-Arques et al., 2016). *Prevotella* is the predominant bacterial genus in the human gut microbiome (Gálvez et al., 2020). *P. copri* has a proinflammatory function in several diseases that involves enhancing resistance to host-derived ROS and producing the redox protein thioredoxin (Hofer, 2014). However, in a cohort of 276 Chinese patients, *P. acnes* abundance was increased whereas that of *P. copri* was decreased in GC tissue (Liu et al., 2019a). Given these incongruent findings, additional studies are needed to elucidate the role of *P. copri* in gastric carcinogenesis.

The gut microbiome can modulate the human immune system (Schluter et al., 2020), and the same is likely true for the gastric microbiome. A study involving 64 patients with GC found that BDCA2 + pDCs and Foxp3 + Tregs were more abundant in tumoral and peritumoral tissues than in normal tissues (Ling et al., 2019). Moreover, *Stenotrophomonas* and *Selenomonas* abundance was positively correlated with the number of BDCA2 + pDCs and Foxp3 + Tregs, respectively, whereas *Comamonas* and *Gaiella* abundance was negatively correlated with BDCA2 + pDCs and Foxp3 + Tregs numbers, respectively (Ling et al., 2019). Additionally, a serum microbiome analysis found that *Comamonas* was enriched in healthy controls relative to GC patients (Dong et al., 2019). This suggests that alterations in the gastric microbiome profile can modulate immune cell populations that contribute to the establishment of an immunosuppressive microenvironment (Ling et al., 2019). pDCs and Tregs have been reported to suppress antitumor immunity, enabling tumor cell evasion of immune surveillance mechanisms (Huang et al., 2014). There is little known about the interaction between *Stenotrophomonas* and human DCs, although *Stenotrophomonas* achieved immune escape by circumventing phagocytosis and stimulated the expression of TNF-α and IL-12 by DCs to promote inflammation (Roscetto et al., 2015). More research is required to clarify the mechanism of microbiome-driven immune modulation.

The increased abundance of *Fusobacterium* in patients with GC was shown to have diagnostic value (Hsieh et al., 2018). *Fusobacterium nucleatum* predicted worse prognosis in Lauren’s diffuse-type GC but not in intestinal-type GC (Boehm et al., 2020). Moreover, *Fusobacterium* sp. infection was positively correlated with the tumor-infiltrating lymphocytes and p53 expression in GC tissues (Nie et al., 2021). *F*. *nucleatum* has been detected in various diseases including appendicitis, inflammatory bowel disease, pancreatic cancer, and colorectal cancer (Swidsinski et al., 2011; Castellarin et al., 2012; Shaw et al., 2016; Del Castillo et al., 2019). However, the pathogenic mechanisms of *F*. *nucleatum* in GC are unknown. In colorectal cancer, interaction between the *F*. *nucleatum* adhesin FadA and E-cadherin of epithelial cells activated β-catenin and the Wnt signaling pathway (Rubinstein et al., 2013). *F*. *nucleatum* also drives the activation of NF-κB signaling and increased the expression of proinflammatory cytokines such as IL-1β, IL-6, IL-8, and TNF (Kostic et al., 2013; Brennan and Garrett, 2019). Outer membrane vesicles of *F*. *nucleatum* can interact with host epithelial cells to induce inflammatory responses and epithelial-mesenchymal transition (EMT) (Hashemi Goradel et al., 2019). Another *F*. *nucleatum* adhesin, fibroblast activation protein (Fap)2, can bind to T cell immunoglobulin and immunoreceptor tyrosine-based inhibitory motif domain (TIGIT) receptor expressed by natural killer cells to block antitumor immune responses (Gur et al., 2015). A similar mechanism may be employed by *F*. *nucleatum* to promote the development of GC.

#### Microbial Metabolites of Non-*H. pylori* Bacteria That Promote Gastric Carcinogenesis

Nitrosating agents play an important role in gastric carcinogenesis (Correa, 1992). Humans are exposed to N-nitroso compounds (NOCs) from exogenous sources such as processed meat, smoked fish, and certain vegetables as well as from endogenous synthesis (Tsugane and Sasazuki, 2007). It is well-established that NOCs have carcinogenic effects. Epidemiologic studies have shown that patients with GC have higher NOC levels than healthy subjects (Walters et al., 1982; Xu et al., 2015). However, food contains only small amounts of nitrite, which is mainly derived from the reduction of nitrate to nitrite by oral bacteria in saliva (Hyde et al., 2014; Burleigh et al., 2018). After entering stomach, nitrite is converted to NOCs, which could be inhibited by ascorbic acid (Kobayashi, 2018). Several bacteria such as *Veillonella*, *Clostridium*, *Haemophilus*, *Staphylococcus*, *Neisseria*, *Lactobacillus*, and *Nitrospirae* contribute to gastric carcinogenesis by stimulating the production of NOCs (Wang et al., 2016; Zhang et al., 2019). Nitrosating or nitrate−reducing bacteria were found to be more abundant in GC patients than control subjects, although the difference between the 2 groups was not statistically significant (Jo et al., 2016). Higher nitrate and nitrite reductase activities associated with the microbiome were observed in GC than in chronic gastritis (Ferreira et al., 2018). Subtotal gastrectomy significantly altered the gastric microbiome profile and decreased the level of nitrate and nitrite reductases and the expression of genes involved in nitrosation (Tseng et al., 2016). These results indicate that changes in the gastric environment that reduce acid secretion leading to the growth of NOC-producing bacteria, thereby increasing the risk of gastric carcinogenesis.

The abundance of lactic acid bacteria was shown to be increased in patients with GC (Yu et al., 2017). Although a large number of literatures have discussed the protective effects of lactic acid bacteria against GC, it may increase the risk of GC through multiple mechanisms including increased production of ROS, NOCs, and lactate as well as induction of EMT and immune tolerance. *In vitro* and *in vivo* experiments have demonstrated that lactic acid bacteria stimulate the generation of ROS that cause DNA damage and enhance the formation of NOCs that induce mutagenesis, angiogenesis, and protooncogene expression and inhibit apoptosis (Jones et al., 2012; Vinasco et al., 2019). Lactic acid bacteria also increase the production of lactate; the levels of L- and D-lactate and lactate dehydrogenase were higher in patients with gastric carcinoma than in those with gastric ulcers and healthy controls (Armstrong et al., 1984). Lactate is a source of energy for cancer cells (Faubert et al., 2017) and plays a regulatory role in various aspects of carcinogenesis including tumor angiogenesis, immune escape, tumor cell migration, and metastasis (San-Millán and Brooks, 2017). Lactic acid bacteria can promote EMT – a process characterized by the loss of adherence junctions and polarity in epithelial cells and emergence of mesenchymal phenotypes that contribute to tumor invasion, migration, and metastasis (Yeung and Yang, 2017) – by inducing a state of multipotency (Vinasco et al., 2019). Finally, lactic acid bacteria promote the establishment of immune tolerance, allowing colonization by other carcinogenic bacteria (van Baarlen et al., 2009; Vinasco et al., 2019).

The role of the extragastric microbiome in gastric carcinogenesis has also been investigated using animal models. Colonization of mice with enterohepatic *Helicobacter* species prior to *H. pylori* infection influenced the severity of *H. pylori*-induced gastric lesions, suggesting that the interaction between *H. pylori* and the extragastric microbiome is an important mechanism underlying gastric carcinogenesis (Lemke et al., 2009; Ge et al., 2011).

## Role of Bacteria in the Treatment of *H. pylori* Infection

Eradication of *H. pylori* infection is an effective strategy for reducing the risk of GC. The Maastricht V/Florence Consensus Report recommends PPI-clarithromycin-containing triple therapy as a first-line treatment for this purpose (Malfertheiner et al., 2017). Several studies have demonstrated that antibiotic treatment of *H. pylori* infection altered the composition of the gastric microbiome (Li et al., 2017; Zhang et al., 2019). However, given the increased rates of antibiotic resistance in *H. pylori*, there is an urgent need for novel *H. pylori* eradication strategies.

The Food and Agriculture Organization of the United Nations and the WHO define probiotics as “live microorganisms which when administered in adequate amounts confer a health benefit on the host” (Hill et al., 2014). Some probiotics prevent antibiotic-induced adverse effects, increase *H. pylori* eradication rates, and reduce fluctuations in the gut microbiome profile (Oh et al., 2016). One of the most widely studied probiotics is *Lactobacillus*, which is widely used in food production and clinical practice to balance the microbial ecosystem in the human gastrointestinal tract (Seddik et al., 2017). Some strains of *Lactobacillus* mitigate *H. pylori* infection by preventing its adhesion to epithelial cells, producing bacteriocins or organic acids, and suppressing mucosal inflammation (Gotteland et al., 2006; Yang and Sheu, 2012; Sakarya and Gunay, 2014). *Lactobacillus acidophilus* and *Lactobacillus bulgaricus* were shown to decrease the adhesion of *H. pylori* to gastric mucosal cells (Song et al., 2019), and *L*. *bulgaricus* inhibited IL-8 production by mucosal cells by modulating the TLR4/IκBα/NF-κB pathway (Song et al., 2019). *Lactobacillus* supplementation was effective in eradicating *H. pylori* infection in patients with chronic gastritis (Francavilla et al., 2008; Fang et al., 2019; Yu et al., 2019). Multi-strain probiotics can also increase *H. pylori* eradication rates and prevent adverse events (McFarland et al., 2016): a *Lactobacillus*- and *Bifidobacterium*-containing probiotic mixture was shown to exert beneficial effects against *H. pylori*, with a low incidence of side effects (Wang et al., 2013); and a combination of *Bifidobacterium infantis*, *L*. *acidophilus*, *Enterococcus faecalis*, and *Bacillus cereus* enhanced host immunity and reduced inflammation in GC patients who underwent gastrectomy (Zheng et al., 2019).

## Role of Bacteria in the Treatment of GC

Conventional treatments for GC including surgery, chemotherapy, and radiation therapy are not very effective. Therefore, novel treatment strategies are needed. Although bacteria were traditionally regarded as carcinogenic, there is now available evidence for their anticancer properties. The role of the microbiome in cancer treatment was proposed as early as 1867, when it was reported that infection with *Streptococcus pyogenes* caused cancer remission in a patient (Sawant et al., 2020). Bacteria exert anticancer effects via multiple mechanisms including (i) colonizing tumors, (ii) releasing substances, (iii) suppressing nutrients required for tumor metabolism and proliferation, (iv) serving as a vehicle for anticancer drugs delivery, (v) forming biofilms, and (vi) enhancing host immunity (Soleimanpour et al., 2020; Yaghoubi et al., 2020).

*Helicobacter pylori* ribosomal protein (HPRP)-A1 and its enantiomer HPRP-A2 are 15-mer cationic peptides that are derived from the N terminus of the ribosomal protein L1 of *H*. *pylori* (Mai et al., 2015). HPRP-A1 and HPRP-A2 show strong antimicrobial and anticancer activities. HPRP-A1 is a membrane-active peptide that can disrupt the tumor cell membrane, and is thus often used to aid the delivery of other drugs to cancer cells (Zhao et al., 2015b). The KLA peptide exerts anticancer effects by inducing apoptosis via disruption of mitochondrial membranes, but has low membrane penetration (Hu et al., 2018). HPRP-A1 facilitates the entry of KLA peptide into cancer cells, which localizes to mitochondrial membranes to promote tumor cell death (Hao et al., 2019). HPRP-A2 induces the apoptosis of GC cells by enhancing ROS production; activating caspase-3, caspase-8 and caspase-9; reducing mitochondrial membrane potential; and causing cell cycle arrest in the G1 phase (Zhao et al., 2015a).

Besides HPRP-A1 and HPRP-A2, ieodoglucomides B – a glycolipopeptide isolated from the marine bacterium *Bacillus licheniformis* – has been demonstrated to have cytotoxic activity against stomach cancer cell lines (Tareq et al., 2012). Additionally, FW523-3 – a lipopeptide compound isolated from the culture broth of the marine bacterium *Micromonospora chalcea* – was shown to inhibit the proliferation of multiple cancer cells types including GC cells (Xie et al., 2011).

## Conclusion

Growing evidence suggests the relationship between gastric microbiome and the development of GC. Changes of the gastric microbiome across different disease stages have been described. This is probably owed to the interactions between the gastric microbiota, environment, and host immune response. Although numerous studies investigating the carcinogenic mechanisms of *H. pylori* have been performed, limited progress has been made regarding the definite role of non-*H. pylori* in the development of GC over the past decades. Currently, only few studies have focused on the possible carcinogenic roles of non-*H. pylori* and their metabolites, including induction of inflammatory response, modulation of immune response, induction of DNA damage, and promotion of EMT. Therefore, further investigations are required to elucidate the detailed carcinogenic mechanisms of gastric microbiome to provide novel insights for the diagnosis, prevention, and treatment of GC.

## Author Contributions

ZL and FJ designed the review and revised the manuscript. JY performed the literature search and wrote the manuscript. XZ performed the literature search and analyzed the literature. XL prepared the manuscript figure and revised the manuscript. All authors approved the final version of the manuscript.

## Conflict of Interest

The authors declare that the research was conducted in the absence of any commercial or financial relationships that could be construed as a potential conflict of interest.
